# Determinants of Self-monitoring of Blood Glucose in Iranian Children and Adolescents with Type 1 Diabetes

**DOI:** 10.5812/ijem-138377

**Published:** 2023-11-19

**Authors:** Farimah Fayyaz, Sepehr Khosravi, Asieh Mosallanejad, Ozra Tabatabaei-Malazy, Seyed Saeed Hashemi Nazari, Maede Shaghaghi

**Affiliations:** 1 Colorectal Research Center, Iran University of Medical Sciences, Tehran, Iran; 2 Non-communicable Diseases Research Center, Endocrinology and Metabolism Population Sciences Institute, Tehran University of Medical Sciences, Tehran, Iran; 3 Imam Hosein Medical Center, Shahid Beheshti University of Medical Sciences, Tehran, Iran; 4 Department of Epidemiology, Prevention of Cardiovascular Disease Research Center, School of Public Health and Safety, Shahid Beheshti University of Medical Sciences ,Tehran, Iran; 5 Students Research Committee, School of Medicine, Shahid Beheshti University of Medical sciences, Tehran, Iran

**Keywords:** Diabetes Mellitus, Type 1, Insulin, Blood Glucose Self-monitoring

## Abstract

**Background:**

Type 1 diabetes mellitus (T1DM) is a prevalent chronic disease among children and adolescents, necessitating effective self-monitoring of blood glucose (SMBG) levels. Understanding the determinants and factors influencing SMBG behavior is crucial for optimizing diabetes management in this population.

**Objectives:**

This study aimed to investigate the frequency of SMBG and identify the determinants influencing factors in children and adolescents with T1DM.

**Methods:**

This cross-sectional study was conducted in Tehran, Iran, and included 275 participants selected through simple random sampling from the Gabric Diabetes Education Association. The inclusion criteria comprised children and adolescents aged 3 - 18 years diagnosed with T1DM for at least 6 months who were using analog or neutral protamine Hagedorn (NPH) and regular insulin subcutaneously. Patients using insulin pumps were excluded. Data collection involved an online questionnaire covering demographic information (e.g., age, gender, educational status, and parental occupations) as well as clinical information (number of hypoglycemic episodes, hemoglobin A1C (HbA1C) levels, diabetes duration, insulin regimen, diabetes complications, glucose monitoring practices, hospitalizations, and behavioral characteristics). Statistical analyses, including descriptive statistics, correlation tests, and Poisson regressions, were performed using SPSS software (version 21). A significance level of P-value < 0.05 was considered statistically significant.

**Results:**

The participants had a mean age of 10.00 ± 3.77 years, with 54.2% being males. Most of the participants (87.3%) were schoolchildren, and the mean age of diagnosis was 6.56 ± 3.73 years, with a mean duration of 44.72 ± 36.32 months. Anthropometric investigations revealed mean height, weight, and body mass index (BMI) values of 136.69 ± 21.11 cm, 37.45 ± 15.51 kg, and 18.31 ± 3.55 kg/m^2^, respectively. The majority of participants (93.5%) used insulin pens, and the mean daily insulin dosage was 35.34 ± 22.20 IU. Parents reported consistent glucose level monitoring in 64.7% of cases. The mean HbA1c level was 7.91 ± 1.58%. Factors such as the price and availability of glucometer strips influenced glucose level monitoring. In univariate analysis, only age and HbA1C levels showed a negative correlation; however, parents’ consistent checking showed a positive correlation with the frequency of daily, weekly, or monthly glucose checking.

**Conclusions:**

This study underscores the significance of SMBG in children and adolescents with T1DM. The findings emphasize the critical role of price and availability of glucometers and strips in achieving standard care for T1DM patients.

## 1. Background

Diabetes mellitus (DM) is a prevalent metabolic disorder that affects multiple organs, leading to chronic vascular and non-vascular complications. Among the various types of diabetes, type 1 DM (T1DM) is characterized by the destruction of insulin-secreting pancreatic β cells. According to the 10th edition of the International Diabetes Federation Atlas, T1DM is estimated to affect around 1,200,000 children worldwide (1). In Iran, the prevalence rate of T1DM was reported to be 388.9 per 100,000 individuals in 2019 (2). Individuals with T1DM face an increased risk of various complications. Although insulin administration remains crucial, blood glucose control through self-monitoring plays a pivotal role in preventing diabetes-related complications (3).

Therefore, it is imperative to explore effective methods for maintaining optimal blood glucose levels and identify the factors that influence self-monitoring in patients with diabetes. Despite the well-established benefits of self-monitoring of blood glucose (SMBG), challenges such as limited education, absence of national guidelines, and high monitoring device costs hinder its widespread adoption. In the context of Iran, where the prevalence of T1DM is notable, there is a gap in understanding the frequency and influencing factors of SMBG among children and adolescents.

Numerous studies have demonstrated the significant impact of SMBG on glycemic control, resulting in reduced hemoglobin A1C (HbA1c) levels and a decreased risk of ophthalmic, renal, neural, and cardiovascular complications (4-7). Scientific guidelines currently recommend measuring blood glucose levels four times a day (8). Although several studies have explored the impact of SMBG on glycemic control and its associated benefits and barriers (9, 10), this study’s focus on the specific population of children and adolescents with T1DM in Iran makes it particularly relevant. This study addresses the unique challenges and barriers faced by this demographic in the Iranian context, offering insights that can inform tailored interventions and educational programs.

## 2. Objectives

In 2022, the objective of this study was to assess the frequency of SMBG and identify influencing factors among children and adolescents with T1DM who are members of the Gabric Diabetes Education Association (www.gabric.ir) in Tehran, Iran.

## 3. Methods

### 3.1. Study Design and Population

This cross-sectional study aimed to investigate the frequency of SMBG and its influencing factors among children and adolescents with T1DM in Tehran, Iran. The participants in this study were recruited from the Gabric Diabetes Education Association, a non-governmental organization in Tehran, Iran.

The methodology for participant selection in this study involved employing a simple random sampling technique to ensure the unbiased representation of a subset of the population from the Gabric Diabetes Education Association. To achieve this, a computer-generated random number generator was utilized to select members of the Gabric Diabetes Education Association as the study’s sample. Subsequently, the chosen members were contacted via text messages until a total sample size of 275 patients was attained.

The inclusion criteria for participants were children and adolescents aged 3 - 18 years diagnosed with T1DM for at least 6 months and using analog or neutral protamine Hagedorn (NPH) and regular insulin subcutaneously. Patients who used insulin pumps were excluded from this study. The sample size was calculated using the formula below and information from Fayyaz et al.’s study (7). The alpha level was set at 5%, and the total sample size was determined to be 275 participants.


$$n=\frac{z_{1-\frac{\alpha }{2}}^{2}\times P(1-P)}{d^{2}}$$

P = 75%, α = 5%

### 3.2. Data Collection and Questionnaire

An online questionnaire was administered to all participants via an electronic link to collect the necessary data. The questionnaire comprehensively captured both demographic and clinical information. The demographic section of the questionnaire included questions about age, gender, educational status, and the occupations of the participants’ parents. In addition to demographic details, the questionnaire extensively covered various clinical aspects. These encompassed essential parameters, such as the number of hypoglycemic episodes experienced by the participants, their HbA1C levels, and the duration of their diabetes diagnosis, in addition to anthropometric measurements encompassing height, weight, and body mass index (BMI).

Moreover, the questionnaire delved into crucial aspects related to the participants’ insulin regimen, any complications associated with their diabetes, their practices in monitoring glucose levels, the frequency of hospitalizations, and their behavioral characteristics. By gathering this comprehensive range of information, the questionnaire aimed to provide a thorough understanding of various factors influencing SMBG in children and adolescents with T1DM.

### 3.3. Ethical Considerations

This study was approved by the Ethics Review Board of Shahid Beheshti University of Medical Sciences, Tehran, Iran (code no: IR.SBMU.MSP.REC.1399.757). Ethical principles were strictly adhered to throughout the study. Verbal informed consent was obtained from all participants and their parents or legal guardians. Participant confidentiality was ensured by anonymizing personal information and using assigned codes for data analysis. The right to withdraw from the study at any time without negative consequences on care or services was emphasized. The study design and objectives were transparently communicated to participants, ensuring their full understanding.

### 3.4. Data Analysis

The statistical analysis was conducted using SPSS software (version 21.0; SPSS Inc., IBM Company). Categorical variables were reported as frequencies and percentages; nevertheless, continuous variables were presented as means with their corresponding standard deviations (SD) or medians with interquartile ranges (IQR). Descriptive statistical methods were employed to analyze the collected data.

Negative binomial regression was performed to evaluate the association of the weekly and monthly serum glucose levels checking sessions with age, gender, BMI, diagnosis age, disease duration, insulin regimen, total daily insulin dose, HbA1c level, and diabetes-related hospitalizations. Statistical significance was set at a threshold of P-value < 0.05.

## 4. Results

A total of 275 participants met the inclusion criteria. The mean age of the patients was 10.00 ± 3.77 years, with 54.2% being male. Moreover, 260 patients (94.5%) were located in Tehran, and all patients, except for one case, were living with their families. Regarding educational status, 240 patients (87.3%) were schoolchildren.

Regarding disease diagnosis and duration, the mean age of diagnosis was 6.56 ± 3.73 years, with a mean duration of 44.72 ± 36.32 months. Anthropometric investigations revealed a mean height, weight, and BMI of 136.69 ± 21.11 cm, 37.45 ± 15.51 kg, and 18.31 ± 3.55 kg/m^2^, respectively. Parental occupations were obtained using an online questionnaire, suggesting that the most common paternal occupation was freelancing, with 160 cases (58.2%), and most maternal occupations were stay-at-home mothers, with 216 cases (78.5%) (Table 1). 

**Table 1.** Baseline Patients’ Characteristics

Variables	Mean ± SD	Median	Minimum, Maximum	IQR	No. (%)
**Quantitative**					
**Age (y)**	10.00 ± 3.77	10.00	2, 20	7.00 - 13.00	
**Age of diagnosis (y)**	6.56 ± 3.73	6.00	1, 16	4.00 - 9.00	
**Duration of the disease (mo)**	44.72 ± 36.32	36.00	0.5, 159	16.00 - 66.00	
**Height (cm)**	139.69 ± 21.11	140.00	90, 189	124.50 - 156.00	
**Weight (kg)**	37.45 ± 15.51	35.00	13, 81	24.50 - 49.00	
**Body mass index**	18.31 ± 3.55	17.95	12.62, 31.95	15.42 - 20.39	
**Qualitative**					
**Gender**					
Male					149 (54.2)
Female					126 (45.8)
**Settlement**					
Tehran					260 (94.5)
Other cities					15 (5.5)
**Education**					
School-aged					240 (87.3)
Preschool-aged					35 (12.7)
**Paternal occupation**					
Freelancer					160 (58.2)
Employee					99 (36.0)
Retired					9 (3.3)
Unemployed					6 (2.2)
**Maternal occupation**					
Stay-at-home mothers					216 (78.5)
Employee					37 (13.5)
Freelancer					19 (6.9)
Retired					3 (1.1)
**Insurance**					
Yes					247 (89.8)
No					28 (10.2)

Abbreviations: SD, standard deviation; IQR, interquartile range.

Regarding diabetes, 257 patients (93.5%) used insulin pens, and 18 patients (6.5%) used the NPH-regular regimen. The mean dosage of total daily insulin was 0.608 ± 1.413 IU/kg, with a median of 0.96 IU/kg. In 97 cases (35.3%), parents sometimes checked their daily glucose levels; nevertheless, in 178 cases (64.7%), parents always checked their daily glucose levels. The mean HbA1c level was 7.91 ± 1.58%. Carbohydrate counting for insulin dosage adjustment was used in 198 cases (72.0%). Diabetes-related complications were observed in 7 cases (2.5%) with retinal manifestations, 7 cases (2.5%) with renal manifestations, and 4 cases (1.5%) with diabetic foot ulcer complications. Hospitalization occurred due to diabetic ketoacidosis (DKA) in 82 cases (29.81%) and due to hypoglycemia in 15 cases (5.5%). In 148 cases (53.9%), glucose levels were checked more than four times daily. The mean frequency of glucose level checks was 27.74 ± 19.69 and 96.26 ± 71.18 weekly and monthly, respectively (Table 2). 

**Table 2.** Diabetes Regimen and Diabetes-Related Complications

Variables	Mean ± SD	Median	Minimum, Maximum	IQR	No. (%)
**Quantitative**					
**Total daily dosage**	35.34 ± 22.20	30.00	5, 141	18.00 - 46.00	
**HbA1c**	7.91 ± 1.58	7.50	5, 13	7.00 - 9.00	
**Weekly glucose check**	27.74 ± 19.69	28.00	0, 80	10 - 40	
**Monthly glucose check**	96.26 ± 71.18	100.00	0, 250	29.00 - 150.00	
**Qualitative**					
**Insulin regimen**					
Pens					257 (93.5)
NPH-regular					18 (6.5)
**Parental daily glucose check**					
Sometimes					97 (35.3)
Always					178 (64.7)
**Carbohydrate counting**					
Yes					198 (72.0)
No					77 (28.0)
**Complications**					
Renal					7 (2.5)
Retinal					7 (2.5)
Foot ulcer					4 (1.5)
**Hospitalization for DKA**					
**Yes**					82 (29.8)
Once					71 (25.8)
Twice					9 (3.3)
More than twice					1 (0.8)
**No**					193 (70.2)
**Hospitalization for hypoglycemia**					
**Yes**					15 (5.5)
Once					9 (3.3)
Twice					4 (1.5)
More than twice					2 (0.7)
**No**					260 (94.5)
**Glucose check**					
Less than once weekly					20 (7.3)
Once weekly					7 (2.5)
Once daily					17 (6.2)
2 - 4 times daily					83 (30.2)
More than 4 times daily					148 (53.9)

Abbreviations: SD, standard deviation; IQR, interquartile range; HbA1c, hemoglobin A1C; NPH, neutral protamine Hagedorn; DKA, diabetic ketoacidosis.

The price of glucometer strips (49.4%), unavailability of glucometer strips (40.4%), and the price of the glucometer (33.1%) were the top three most important factors regarding glucose level checking (Tables 3 and 4). Assessing the correlation between weekly and monthly glucose checking frequency revealed a significant association with age, age of diagnosis, using carbohydrate counting, parental glucose checking, BMI, and HbA1c in univariate analysis; however, multivariate analysis showed a significant association only with carbohydrate counting. Tables 5 and 6 show associations between daily, weekly, and monthly glucose-checking sessions with other factors.

**Table 3.** Factors Affecting Glucometer Check

Variables	Disagree, No. (%)	Agree, No. (%)	Strongly Agree, No. (%)
**Price of glucometer**	86 (31.3)	98 (35.6)	91 (33.1)
**Pain**	108 (39.3)	98 (35.6)	69 (25.1)
**Strip availability**	100 (36.4)	64 (23.3)	111 (40.4)
**Doubt regarding usefulness**	191 (69.5)	34 (12.4)	50 (18.2)
**Peer pressure **	121 (44.0)	65 (23.6)	89 (32.4)
**Not receiving patient education**	193 (70.2)	26 (9.5)	56 (20.4)
**Not enough time**	190 (69.1)	44 (16.0)	41 (14.9)
**Glucometer availability**	158 (57.5)	54 (19.6)	63 (22.9)
**Forgetfulness**	181 (65.8)	48 (17.5)	46 (16.7)
**Fear of perception**	137 (49.8)	71 (25.8)	67 (24.4)
**Price of strips**	85 (30.9)	53 (19.3)	137 (49.9)
**Sufficiency of clinical measurement**	230 (83.6)	19 (6.9)	26 (9.5)

**Table 4.** Obstacles Regarding Glucose Level Check ^a^

	Mean ± SD	Median
**Pain**	3.70 ± 2.38	3.00
**Strip availability**	2.88 ± 2.31	2.00
**Not receiving patient education**	5.13 ± 2.39	7.00
**Not enough time**	5.02 ± 2.29	6.00
**Forgetfulness**	4.48 ± 2.32	5.00
**Fear of perception**	4.55 ± 2.34	5.00
**Price of strips**	2.84 ± 2.30	1.00

Abbreviations: SD, standard deviation.

^a^ A reverse scoring system is used, meaning lower measurements are more important.

**Table 5.** Assessment of the Correlation Between Daily Glucose Checking Sessions and Other Factors Using Negative Binomial Regression

Variables	Daily					
	Multivariate			Univariate		
	PR	Sig.	95% CI	PR	Sig.	95% CI
**Age**	1.002	0.937	0.961 - 1.044	0.978	0.024	0.959 - 0.997
**Being male**	0.931	0.350	0.802 - 1.081	0.934	0.354	0.808 - 1.079
**Being a student**	1.075	0.574	0.835 - 1.385	0.941	0.586	0.757 - 1.170
**Age of diagnosis**	0.971	0.163	0.933 - 1.012	0.979	0.060	0.959 - 1.001
**Duration of the disease**	0.999	0.498	0.995 - 1.002	0.999	0.165	0.997 - 1.001
**Use of insulin regular/NPH**	0.929	0.664	0.665 - 1.297	0.914	0.584	0.662 - 1.262
**Using carbohydrate count**	1.056	0.563	0.877 - 1.272	1.163	0.085	0.980 - 1.381
**Parents always checking**	1.072	0.443	0.898 - 1.280	1.182	0.034	1.013 - 1.380
**BMI**	1.001	0.949	0.977 - 1.025	0.987	0.232	0.967 - 1.008
**HbA1c**	0.964	0.156	0.917 - 1.014	0.961	0.097	0.916 - 1.007
**Hospitalization for DKA**	1.151	0.128	0.960 - 1.380	1.118	0.173	0.952 - 1.312
**Hospitalization for hypoglycemia**	0.788	0.199	0.548 - 1.133	0.861	0.394	0.610 - 1.215

Abbreviations: CI, confidence interval; Sig, significance; PR, prevalence ratio; NPH, neutral protamine Hagedorn; BMI, body mass index; HbA1c, hemoglobin A1C; DKA, diabetic ketoacidosis.

**Table 6.** Assessment of the Correlation Between Weekly and Monthly Glucose-Checking Sessions and Other Factors Using Negative Binomial Regression

Variables	Multivariate			Univariate		
	PR	Sig.	95% CI	PR	Sig.	95% CI
**Weekly**						
**Age**	0.959	0.086	0.914 - 1.006	0.938	< 0.001	0.913 - 0.964
**Being male**	1.008	0.938	0.832 - 1.221	1.044	0.681	0.850 - 1.283
**Being a student**	1.187	0.313	0.851 - 1.657	0.832	0.249	0.608 - 1.138
**Age of diagnosis**	0.964	0.148	0.918 - 1.013	0.944	< 0.001	0.916 - 0.972
**Duration of the disease**	1.002	0.458	0.997 - 1.006	0.998	0.175	0.995 - 1.001
**Use of insulin regular/NPH**	0.771	0.222	0.507 - 1.171	0.784	0.279	0.504 - 1.218
**Using carbohydrate count**	1.370	0.009	1.081 - 1.737	1.578	< 0.001	1.255 - 1.984
**Parents always checking**	1.121	0.318	0.896 - 1.401	1.356	0.005	1.099 - 1.674
**BMI**	0.995	0.759	0.965 - 1.027	0.971	0.044	0.945 - 0.999
**HbA1c**	0.914	0.007	0.857 - 0.976	0.904	0.003	0.845 - 0.966
**Hospitalization for DKA**	1.258	0.060	0.991 - 1.598	1.124	0.324	0.891 - 1.417
**Hospitalization for hypoglycemia**	0.824	0.382	0.534 - 1.272	1.048	0.839	0.665 - 1.653
**Monthly**						
**Age**	0.968	0.273	0.914 - 1.026	0.931	< 0.001	0.902 - 0.961
**Being male**	1.072	0.549	0.853 - 1.347	1.009	0.439	0.866 - 1.394
**Being a student**	1.246	0.280	0.837 - 1.855	0.805	0.243	0.560 - 1.159
**Age of diagnosis**	0.946	0.057	0.893 - 1.002	0.939	< 0.001	0.907 - 0.971
**Duration of the disease**	0.999	0.848	0.994 - 1.005	0.997	0.116	0.994 - 1.001
**Use of insulin regular/NPH**	0.780	0.327	0.475 - 1.281	0.767	0.306	0.461 - 1.275
**Using carbohydrate count**	1.404	0.017	1.062 - 1.857	1.631	< 0.001	1.251 - 2.127
**Parents always checking**	1.034	0.801	0.798 - 1.340	1.312	0.030	1.027 - 1.677
**BMI**	0.982	0.341	0.947 - 1.019	0.961	0.014	0.930 - 0.992
**HbA1c**	0.938	0.109	0.867 - 1.014	0.915	0.027	0.846 - 0.990
**Hospitalization for DKA**	1.165	0.297	0.874 - 1.553	1.040	0.775	0.794 - 1.362
**Hospitalization for hypoglycemia**	0.778	0.344	0.463 - 1.307	0.970	0.910	0.572 - 1.645

Abbreviations: CI, confidence interval; Sig, significance; PR, prevalence ratio; NPH, neutral protamine Hagedorn; BMI, body mass index; HbA1c, hemoglobin A1C; DKA, diabetic ketoacidosis.

## 5. Discussion

Type 1 diabetes mellitus is a prevalent chronic disease among children and adolescents, accounting for 5 - 10% of all diabetes cases (8). The global burden of diabetes is projected to rise significantly, with an estimated 438 million patients by 2030, including approximately 1.2 million children and adolescents (652,000 children aged under 15 years) worldwide (11). In Iran alone, more than 50,000 young individuals have been diagnosed with diabetes (12). The impact of diabetes is far-reaching, leading to various complications, such as DKA, hypoglycemia, cardiovascular disorders, ophthalmic disorders, renal and neural disorders, cerebrovascular accidents, severe disabilities, and premature death (13-15). Notably, proper education on diabetes plays a critical role in preventing complications, as patients without adequate knowledge are four times more likely to develop complications. Implementing educational measures can reduce complications of chronic diseases by 80% (16, 17).‬‬‬‬‬‬‬‬‬‬‬‬‬‬‬‬‬‬‬‬‬

The American Diabetes Association (ADA) recommends a standard of care for T1DM that involves the administration of multiple daily injections using basal-bolus regimens. This regimen consists of 1 - 2 injections of long-acting insulin, along with rapid-acting insulin for meals and snacks. In addition, SMBG levels play a vital role in ensuring adherence to this standard of care (18). Effective self-management is a cornerstone of diabetes control (19). In the context of diabetes, self-management encompasses daily insulin administration, blood glucose monitoring and maintenance, dietary care, physical activity, embracing a healthy lifestyle with diabetes, and preventing complications (20, 21). Although numerous monitoring approaches have been evaluated for their potential in blood glucose control, the optimal method remains a subject of debate (22).

Effective diabetes management relies on self-care, including monitoring blood glucose levels (19). However, despite the importance of this practice, it can be underused due to confusing guidelines (22). This study explored the factors that influence blood-glucose monitoring in children and adolescents with T1DM. The findings of this study shed light on the key factors influencing SMBG among children and adolescents with T1DM. The cost, price, and availability of glucometer strips emerged as significant factors influencing self-monitoring practices, consistent with prior research that highlighted the financial burden and cost limitations associated with blood glucose monitoring (10, 23-26). The cost of glucometer strips and the price of glucometers in low to middle-income countries can amount to a significant portion of the lowest-paid government workers’ wages, ranging from 4 to 29.9 days, depending on the coverage of T1DM care by the government (27). This underscores the crucial role of price and availability of glucometers and strips in achieving the standard of care for T1DM patients, as highlighted by the findings of this study. However, the financial aspect of self-monitoring is only one of the factors influencing the standard of care for children and adolescents with T1DM. A randomized controlled trial comparing patients who received an intensive educational program for self-monitoring to those who received routine healthcare education showed significant differences in diabetes self-care and knowledge after 1 and 3 months of follow-up (28).

Furthermore, the ADA has emphasized the need for more recent consensus panels to update recommendations related to SMBG, as the last consensus conference on SMBG was held in 1994 (29). In the present study, the lack of patient education emerged as the most prominent obstacle reported by participants in monitoring their glucose levels. These findings further emphasize the necessity of implementing a standardized education program for patients at the time of diagnosis and potential hospitalization. Previous studies reported that proper education on diabetes plays a critical role in preventing complications, as patients without adequate knowledge are four times more likely to develop complications. Implementing educational measures can reduce complications of chronic diseases by 80% (16, 17). Part of these education programs is for children and adolescents; however, the other part is for parents. Higher parental control and efforts were reported to have an association with better adherence to SMBG, which is in line with the findings of this study (30). Efforts from parents to improve the quality of parent-child interaction have been reported to be associated with better T1DM care (31). Moreover, the current study reveals that patients with a history of hospitalization for DKA, female gender, older age, and longer duration of the disease are more inclined to adhere to monthly and weekly glucose monitoring. The aforementioned findings align with previous studies’ findings and provide valuable insights for identifying the target population for standardized education programs and policy-making (32).

Although the present study provides valuable insights into the influencing factors of SMBG, it is not without limitations. Firstly, the present study has a few limitations that should be acknowledged. Firstly, it was conducted in a specific geographic area, focusing on children and adolescents with T1DM in Tehran. Therefore, the findings might not be applicable to other regions or populations with different characteristics or healthcare systems. Secondly, the study relied on self-reported data, which might be subject to biases, such as recall bias or social desirability bias. Despite the aforementioned limitations, the study adds to the existing literature by identifying key factors influencing SMBG in children and adolescents with T1DM. Further research is needed to address these limitations and expand our understanding of self-management strategies in this population.

### 5.1. Conclusions

This study highlights the importance of SMBG in children and adolescents with T1DM. These findings emphasize the critical role of the price and availability of glucometers and strips in achieving standard care for T1DM patients. With proper education, self-monitoring improves blood sugar control significantly. The findings underscore the value of systematic educational programs that enhance understanding of diabetes, its complications, and simple methods of blood sugar control.

## References

[1] 10th, IDF (2021). IDF Diabetes Atlas..

[2] Bandarian F, Sharifnejad Tehrani Y, Peimani M, Namazi N, Saeedi Moghaddam S, Esmaeili S (2023). National and sub-national burden and trend of type 1 diabetes in 31 provinces of Iran, 1990-2019.. Sci Rep..

[3] Hansen MV, Pedersen-Bjergaard U, Heller SR, Wallace TM, Rasmussen AK, Jorgensen HV (2009). Frequency and motives of blood glucose self-monitoring in type 1 diabetes.. Diabetes Res Clin Pract..

[4] Ahlen E, Pivodic A, Wedel H, Dahlqvist S, Kosiborod M, Lind M (2016). Glycemic control, renal complications, and current smoking in relation to excess risk of mortality in persons with type 1 diabetes.. J Diabetes Sci Technol..

[5] Cleary PA, Orchard TJ, Genuth S, Wong ND, Detrano R, Backlund JY (2006). The effect of intensive glycemic treatment on coronary artery calcification in type 1 diabetic participants of the Diabetes Control and Complications Trial/Epidemiology of Diabetes Interventions and Complications (DCCT/EDIC) Study.. Diabetes..

[6] Lind M, Svensson AM, Kosiborod M, Gudbjornsdottir S, Pivodic A, Wedel H (2014). Glycemic control and excess mortality in type 1 diabetes.. N Engl J Med..

[7] Fayyaz F, Aghamahdi F, Noorian S, Tabatabaei-Malazy O, Qorbani M (2022). Associated factors to insulin adherence in type 1 diabetes in Tehran and Karaj, Iran.. J Diabetes Metab Disord..

[8] Nathan DM, Dcct Edic Research Group (2014). The diabetes control and complications trial/epidemiology of diabetes interventions and complications study at 30 years: overview.. Diabetes Care..

[9] Skeie S, Kristensen GB, Carlsen S, Sandberg S (2009). Self-monitoring of blood glucose in type 1 diabetes patients with insufficient metabolic control: focused self-monitoring of blood glucose intervention can lower glycated hemoglobin A1C.. J Diabetes Sci Technol..

[10] Aghili R, Khamseh ME, Malek M, Yarahmadi S, Farshchi A (2012). Structured self monitoring of blood glucose in Iranian people with type 2 diabetes; A cost consequence analysis.. Daru..

[11] Forouhi NG, Wareham NJ (2019). Epidemiology of diabetes.. Medicine..

[12] Pourabassi A, Kheiry Z, Nouriyengejeh S, Naghavi alhosseini S, Banakar F (2019). Identifying the real needs of diabetic children for a better life and assess the alignment of country research activities with these needs; HI inventor project.. Iran J Diabetes Metab..

[13] Edraki M, Zarei A, Soltanian M, Moravej H (2020). The Effect of Peer Education on Self-Care Behaviors and the Mean of Glycosylated Hemoglobin in Adolescents with Type 1 Diabetes: A Randomized Controlled Clinical Trial.. Int J Community Based Nurs Midwifery..

[14] Moravej H, Abedi S, Ghorbani A, Yazdani N, Amirhakimi A, Ilkhanipoor H (2020). The effect of team self –management training on blood sugar control in children and adolescents with type 1 diabetic.. J Diabetes Nurs..

[15] Patterson C, Guariguata L, Dahlquist G, Soltesz G, Ogle G, Silink M (2014). Diabetes in the young - a global view and worldwide estimates of numbers of children with type 1 diabetes.. Diabetes Res Clin Pract..

[16] Cheraghi F, Mortazavi SZ, Shamsaei F, Moghimbeigi A (2014). Effect of education on management of blood glucose in children with diabetes.. J Nurs Educ..

[17] Khalili A, Aghaei S, Doosti-Irani A, Razavi Z, Cheraghi F (2020). Investigating the relationship between demographic characteristics and dimensions of virtual self-care of diabetic children.. Pajouhan Sci J..

[18] Chiang JL, Kirkman MS, Laffel LM, Peters AL, Type 1 Diabetes Sourcebook A (2014). Type 1 diabetes through the life span: a position statement of the American Diabetes Association.. Diabetes Care..

[19] Powers MA, Bardsley J, Cypress M, Duker P, Funnell MM, Hess Fischl A (2015). Diabetes self-management education and support in type 2 diabetes: A joint position statement of the american diabetes association, the american association of diabetes educators, and the academy of nutrition and dietetics.. Diabetes Care..

[20] Hung LC, Huang CY, Lo FS, Cheng SF (2020). The self-management experiences of adolescents with type 1 diabetes: A descriptive phenomenology study.. Int J Environ Res Public Health..

[21] Ranaei Y, Alhani F, Kazemnejad A, Mehrdad N (2018). The effect of lifestyle modification through E-learning on self-management of patients with diabetes.. J Nurs Educ..

[22] Kaufman FR, Halvorson M, Carpenter S (1999). Association between diabetes control and visits to a multidisciplinary pediatric diabetes clinic.. Pediatrics..

[23] Allen BT, DeLong ER, Feussner JR (1990). Impact of glucose self-monitoring on non-insulin-treated patients with type II diabetes mellitus. Randomized controlled trial comparing blood and urine testing.. Diabetes Care..

[24] Nyomba BL, Berard L, Murphy LJ (2004). Facilitating access to glucometer reagents increases blood glucose self-monitoring frequency and improves glycaemic control: a prospective study in insulin-treated diabetic patients.. Diabet Med..

[25] Nyomba BL, Berard L, Murphy LJ (2002). The cost of self-monitoring of blood glucose is an important factor limiting glycemic control in diabetic patients.. Diabetes Care..

[26] Oki JC, Flora DL, Isley WL (1997). Frequency and impact of SMBG on glycemic control in patients with NIDDM in an urban teaching hospital clinic.. Diabetes Educ..

[27] Abdraimova A, Besancon S, Portocarrero J, Ramaiya K, Dunganova A, Ewen M (2022). Management of type 1 diabetes in low- and middle-income countries: Comparative health system assessments in Kyrgyzstan, Mali, Peru and Tanzania.. Diabet Med..

[28] Romero-Castillo R, Pabon-Carrasco M, Jimenez-Picon N, Ponce-Blandon JA (2022). Effects of a diabetes self-management education program on glucose levels and self-care in type 1 diabetes: A pilot randomized controlled trial.. Int J Environ Res Public Health..

[29] Austin MM (2013). The two skill sets of self-monitoring of blood glucose education: The operational and the interpretive.. Diabetes Spectrum..

[30] DasNandy A, Virge R, Hegde HV, Chattopadhyay D (2023). A review of patent literature on the regulation of glucose metabolism by six phytocompounds in the management of diabetes mellitus and its complications.. J Integr Med..

[31] Nieuwesteeg AM, Pouwer F, van Bakel HJ, Emons WH, Aanstoot HJ, Odink R (2011). Quality of the parent-child interaction in young children with type 1 diabetes mellitus: study protocol.. BMC Pediatr..

[32] Mathew R, Gucciardi E, De Melo M, Barata P (2012). Self-management experiences among men and women with type 2 diabetes mellitus: a qualitative analysis.. BMC Fam Pract..

